# High expression of Lewis^y/b ^antigens is associated with decreased survival in lymph node negative breast carcinomas

**DOI:** 10.1186/bcr1305

**Published:** 2005-07-28

**Authors:** Zahra Madjd, Tina Parsons, Nicholas FS Watson, Ian Spendlove, Ian Ellis, Lindy G Durrant

**Affiliations:** 1 Academic Department of Clinical Oncology, Institute of Infections Immunity and Inflammation, University of Nottingham, City Hospital, Nottingham, UK; 2 Scancell Ltd, BioCity, Nottingham, UK; 3 Department of Histopathology, City Hospital, Nottingham, UK

## Abstract

**Introduction:**

There is sufficient evidence that blood group related Lewis antigens are tumour-associated molecules. The Lewis^y ^and Lewis^b ^antigens are complex carbohydrates that are over-expressed by breast, lung, colon and ovarian cancers. The SC101 mAb is a unique Lewis^y/b ^binding antibody that binds to native and extended Lewis^y ^and Lewis^b ^haptens, displaying no cross reactivity with H type 1, H type 2, Lewis^x ^or normal blood group antigens.

**Methods:**

Immunohistochemical detection of Lewis^y/b ^was performed on 660 formalin-fixed, paraffin embedded breast tumour specimens using a streptavidin-biotin peroxidase technique. Tissue from these patients had previously been included in tissue microarrays. This cohort comprises a well characterized series of patients with primary operable breast cancer diagnosed between 1987 and 1992, obtained from the Nottingham Tenovus Primary Breast Carcinoma Series. This includes patients 70 years of age or less, with a mean follow up of 7 years.

**Results:**

Of the breast carcinomas, 370 of 660 (56%) were negative for Lewis^y/b ^expression, 110 (17%) cases showed a low level of expression (<25% of positive cells) and only 54 cases (8%) showed extensive expression of Lewis^y/b ^(>75% of positive cells). We found significant positive associations between histological grade (p < 0.001), Nottingham Prognostic Index (p = 0.016), tumour type (p = 0.007) and the level of Lewis ^y/b ^expression. There was a significant correlation between the proportion of Lewis^y/b ^positive tumour cells and survival in lymph-node negative patients (p = 0.006).

**Conclusion:**

The unique epitope recognised by SC101 mAb on Lewis^y/b ^hapten is over-expressed on breast tumour tissue compared with normal breast. In this large series of invasive breast cancers, higher expression of Lewis^y/b ^was more often found in high grade and poor prognosis tumours compared to good prognosis cancers. Moreover, in lymph node negative breast carcinomas, over-expression of Lewis^y/b ^hapten was associated with significantly decreased patient survival.

## Introduction

Blood group related antigens are frequently altered in association with neoplastic transformation [1-3]. Lewis blood group antigens Lewis^a ^and Lewis^b^, and their positional isomers Lewis^x ^and Lewis^y^, are carbohydrate antigens. Lewis^y ^antigen is a difucosylated oligosaccharide with the chemical structure Fucα1→2Galβ1→4[Fucα1→3]GlcNAcβ1→R, which belongs to the A, B, H, Lewis blood group family. The Lewis^y ^antigen is expressed predominately during embryogenesis, and in adults expression is restricted to granulocytes and epithelial surfaces [4]. Over-expression of Lewis^y ^has been shown in the majority of cancer cells derived from epithelial tissues, however, including breast, ovary, pancreas, prostate, colon and non-small cell lung cancers [5], either at the plasma membrane as a glycolipid or linked to surface receptors (e.g. of the E-rb-B family) [6].

The SC101/29 mAb is a unique Lewis^y/b ^binding antibody that recognises both Lewis^y ^and Lewis^b ^haptens [7]. It shows no cross reactivity with H type 1 or H type 2, Lewis^x ^or normal blood group antigens. This antibody binds strongly to a wide range of tumour tissues, including colon, gastric and ovarian and it may also show strong reactivity with a range of normal tissue expressing either Lewis^y ^or Lewis^b^.

To our knowledge, there has been no previous study examining whether there is any prognostic significance of Lewis^y/b ^expression in breast carcinoma. It was, therefore, of interest to determine the expression of Lewis^y/b ^in a large series of breast cancer tissue arrays using the SC101 mAb. Expression of Lewis^y/b ^was then associated with clinicopathological parameters and patient outcomes.

## Materials and methods

### Patients and tumour characteristics

The study group consisted of 660 primary operable invasive breast carcinomas from patients aged 27 to 70 years (median 54 years) diagnosed from 1987 to 1992 and obtained from the Nottingham Tenovus Primary Breast Carcinoma Series. Patient characteristics, including age and menopausal status, along with information on local, regional and distant recurrence and survival were retrieved from a prospectively maintained database. Patients and tumour characteristics are shown in Table 1. Patients were followed up at 3 month intervals initially, then every 6 to 12 months for a median period of 83 months, with a mean survival of 77 months (1 to 151 months). Ethical approval was granted by the Nottingham Research Ethics Committee.

This is a well characterized series of primary operable breast cancers treated in a uniform manner, and has been used to study a wide range of potential prognostic factors and markers. Tumour characteristics, including histological grade [8], tumour type [9], vascular invasion [10], menopausal status [11], tumour size, lymph node stage and Nottingham Prognostic Index [12] are routinely assessed and recorded in a prospectively maintained database. The Nottingham Prognostic Index (NPI) score was calculated for each patient based on the following equation:

NPI = 0.2 × tumour size (cm) + grade (1–3) + lymph node stage (1–3)

This index predicts survival of patients with invasive breast cancer and is used clinically to define three groups with either a good (NPI =≤ 3.4), moderate (3.41 < NPI ≤ 5.4) or poor (NPI > 5.4) prognosis according to the score obtained. Patient management was based on tumour characteristics by NPI and hormone receptor status. Patients with an NPI score ≤ 3.4 received no adjuvant therapy, those with a NPI score >3.4 received tamoxifen if estrogen receptor (ER) positive (± Zoledex if pre-menopausal) or classical cyclophosphamide, methotrexate and 5-fluorouracil (CMF) if ER negative and fit enough to tolerate chemotherapy.

### Construction of the tissue microarray blocks

At the time of resection, all tumours were managed in a standardised fashion, with immediate incision and fixation in neutral buffered formalin to minimize any diffusion problems, followed by processing through to embedding in paraffin wax. Breast cancer tissue microarrays were prepared as described previously [13]. Microarray samples with a diameter of 0.6 mm were punched from selected regions of each 'donor' block using a manual Tissue Arrayer (Beecher Instruments, Sun Prairie, WI, USA), and precisely arrayed into a new recipient paraffin block. These tissue microarray blocks were constructed in triplicate, at a density of 100 cores per block.

### Immunohistochemistry

The SC101 mAb binds to native and extended Lewis^y ^and Lewis^b ^haptens [7]. Tissue microarray sections 4 μm thick were cut and immunohistochemical staining was performed using a strepavidin-biotin complex (ABC) method as described previously [14]. Microwave pre-treatment was performed in citrate buffer (pH 6.0) for 10 minutes at high power followed by 10 minutes at low power to retrieve antigenicity. The primary antibody SC101 was incubated on the slides for 1 h, at an optimal dilution found to be 1:1000 (stock concentration 1.1 mg/ml). Negative controls, consisting of normal swine serum instead of primary antibody, confirmed the specificity of the staining. Sections from a colon carcinoma previously determined to express Lewis^y/b ^were used as positive control.

### Evaluation of immunostaining

**Figure 1:**
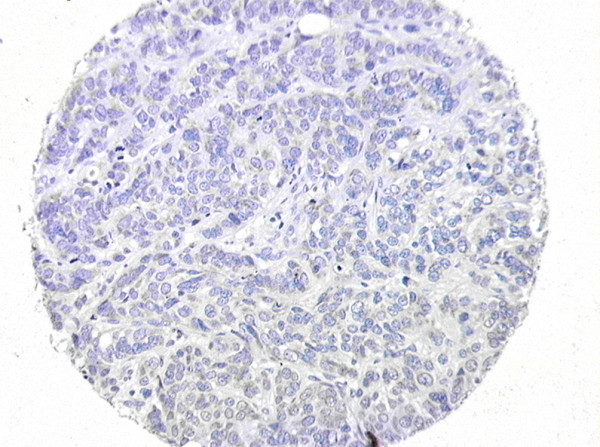
Tissue microarray core demonstrating tumour with absent Lewis^y/b ^expression. Fifty-six percent of breast tumours were entirely negative for Lewis^y/b ^expression using SC101 monoclonal antibody.

**Figure 2:**
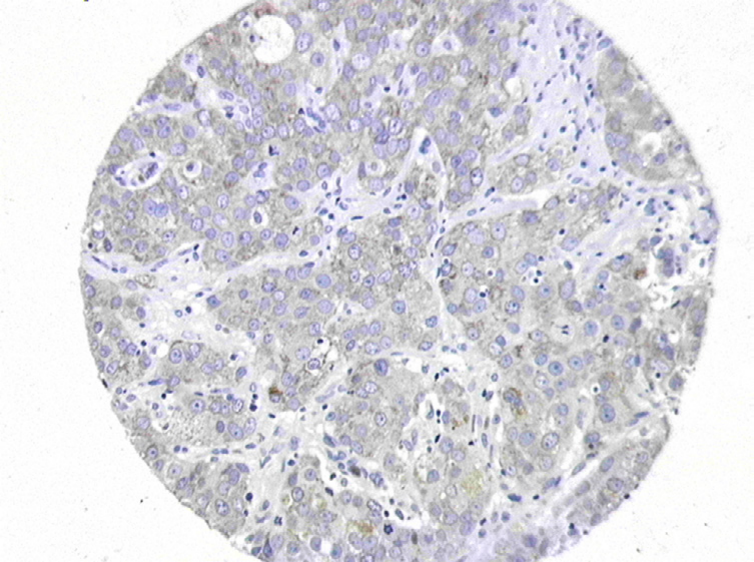
Tissue microarray core demonstrating tumour with weak Lewis^y/b ^expression. Seventy percent of breast tumours showed weak expression of Lewis^y/b ^using SC101.

**Figure 3:**
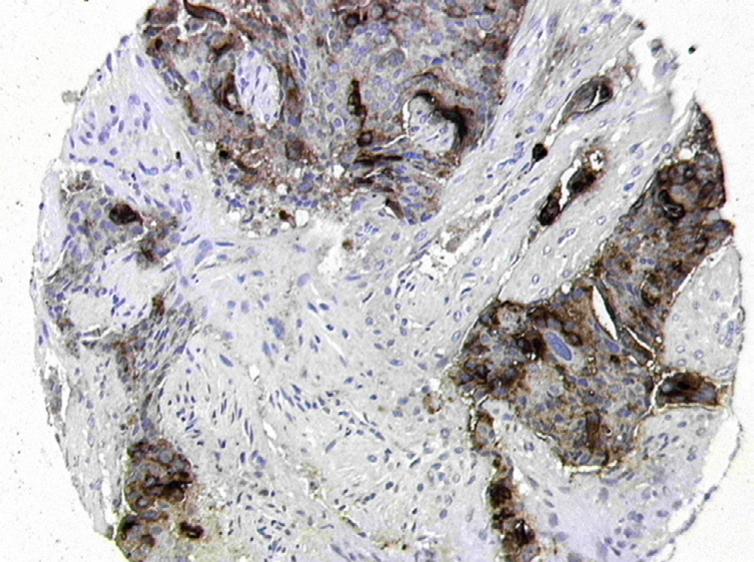
Tissue microarray core demonstrating tumour with moderate Lewis^y/b ^expression. Thirteen percent of tumours showed moderate expression of Lewis^y/b^.

**Figure 4:**
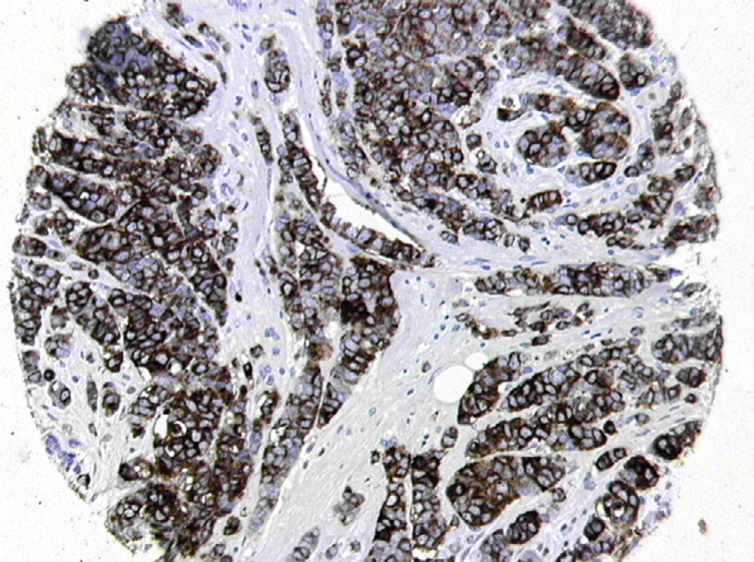
Tissue microarray core demonstrating tumour with strong Lewis^y/b ^expression. Fourteen percent of tumours demonstrated strong expression of Lewis^y/b^.

All immunostained tissue arrays were evaluated using a semi-quantitative system (by ZM) after a series was examined on a double-headed microscope blinded to patients' outcome and other clinical and pathological parameters. The obtained results were confirmed by two observers (ZM and IOE) using a multi-headed microscope and, in difficult cases, a consensus was achieved. Two features were assessed, the intensity of staining and the percentage of cells stained. The intensity of the immunostaining was classified into four categories: 0, no immunostaining present (as shown in Fig. 1); 1, weak immunostaining (Fig. 2); 2, moderate immunostaining (Fig. 3); and 3, strong immunostaining (Fig. 4). The percentage of positive cells at each intensity was also assessed semi-quantitatively, then classified into four groups as 1 (<25% positive cells), 2 (25% to 50% positive cells), 3 (51% to 75% positive cells) or 4 (>75% positive cells). In addition, the histochemical score (H score) of immunoreactivity was obtained by multiplying the intensity and percentage scores [15]. The histochemical scores were subgrouped into three groups of equal range for analysis, and a score of <100 was considered weak, 100 to 200 as moderate and 201 to 300 as strong.

### Statistical analysis

Statistical analysis of data was performed using the SPSS package (version 11 for Windows; SPSS, Chicago, IL, USA). The significance of associations was determined by means of the Pearson R test and/or Pearson chi-square tests. To give sizes of effect and to look at the independence of effects, the percentage of Lewis^y/b ^positive cells reclassified as a binary outcome (high, >25% positive cells; low, <25% positive cells) and effects of clinicopathological parameters were assessed using multiple logistic regression to give adjusted odds ratios and 95% confidence intervals.

In survival analysis, Kaplan-Meier curves were derived and the statistical significance of differences in survival between groups with different Lewis^y/b ^expression was determined using the log-rank test. Survival was censored if the patients were still alive at the time of data analysis, or at the time of death for patients who died from an unrelated cause. Multivariate Cox regression analysis was used to evaluate the independent prognostic effect of variables on overall survival. P-values of <0.05 were identified as statistically significant.

## Results

### Level of expression of Lewis^y/b ^on breast carcinomas

Of the breast carcinomas, 370 out of 660 (56%) were entirely negative for Lewis^y/b ^expression (Fig. 1). A variable percentage of positively staining tumour cells was observed among the remaining 290 breast carcinomas; 110 (17%) cases showed a low level of expression (<25% of positive cells) and only 54 cases (8%) showed extensive expression of Lewis^y/b ^(>75% of positive cells) on the cell membrane (Table 2). The staining observed was predominantly localised to the cell membrane and cytoplasm. No nuclear staining was observed.

Similarly, with regard to the intensity of staining, 17% (106) showed weak expression of Lewis^y/b^, whereas 89 (13%) and 95 (14%) of cases showed moderate and strong staining, respectively (Figs 2, 3, 4; Table 3).

### Association of Lewis^y/b ^expression with clinicopathological characteristics

A frequency histogram for the percentage of positive cells stained demonstrated that the mean value was 25%. This was therefore chosen as an appropriate cut-off for subsequent analysis (data not shown). Tumours were therefore divided into those with <25% versus those with >25% positive cells stained based on the distribution of staining results.

A significant positive relationship was found between the percentage of Lewis^y/b ^positive tumour cells and histological grade of invasive tumours (p < 0.001; Table 4); a higher percentage of Lewis^y/b ^positive tumour cells were identified more frequently in histological grade 3 tumours compared to grade 1 lesions. The odds ratio for higher percentage of Lewis^y/b ^in those with a poor histological grade compared with those with a well differentiated tumour was 2.509 (95% confidence interval, 1.51 to 4.16) (Table 5).

A higher percentage of positive tumour cells was also identified more frequently in tumours from patients in the poor prognosis NPI group (NPI > 5.4) compared with those in the good prognosis group (NPI ≤ 3.4, p < 0.016), with an odds ratio of 1.99 (1.13 to 3.5) (Table 5). In addition, a higher proportion of Lewis ^y/b ^positive tumour cells was seen more frequently in poor prognosis tumour types (ductal/ NST (No special type)), solid lobular, lobular mixed or mixed NST and lobular cancers) compared to the excellent prognosis tumour types (tubulo-lobular, tubular, mucinous and invasive cribriform) (p < 0.007), with an odds ratio of 3.12 (1.07 to 9.11) (Table 5).

There was a positive association between the observed intensity of expression of Lewis^y/b ^and histological tumour grade (p = 0.009). No association was found, however, between intensity of expression and NPI or tumour type. Similarly, we could not find any association between either intensity of expression or the percentage of positive cells and the vascular invasion status, local recurrence, lymph node stage or menopausal status (Table 4).

### Survival analysis

**Figure 5:**
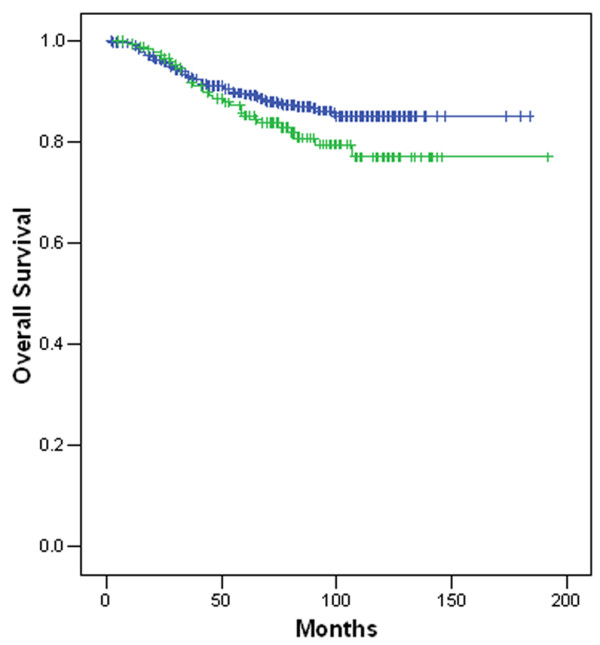
Correlation between the percentage of Lewis^y/b ^positive tumour cells and overall survival (n = 660). Kaplan-Meier survival analysis. Comparison of breast cancer patients with a low percentage of Lewis^y/b ^positive cells (blue curve: <25% positive cells, n = 480), and patients with >25% positive tumour cells (green curve: n = 180, p = 0.091).

**Figure 6:**
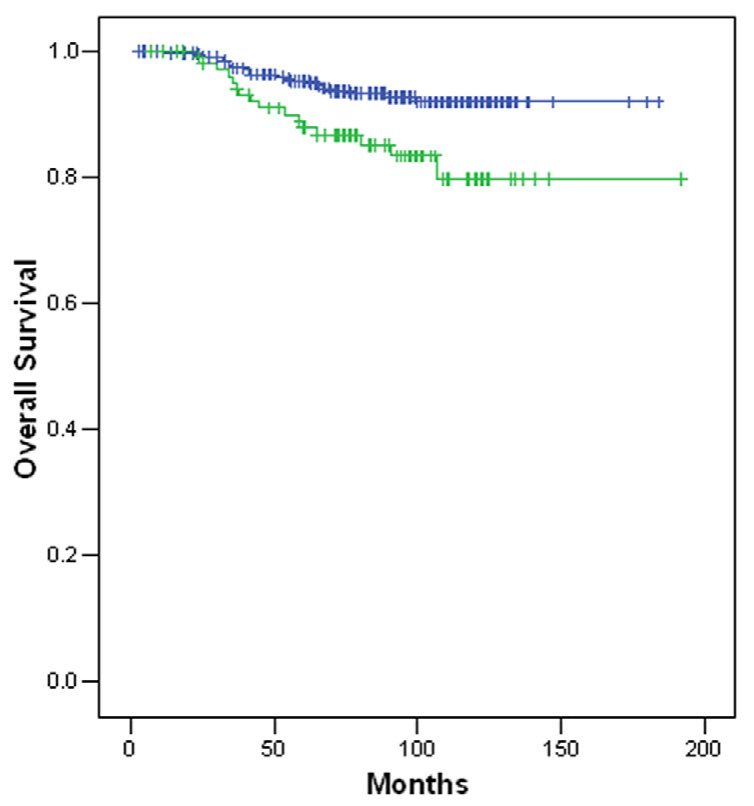
Percentage of Lewis^y/b ^positive cells and overall survival in node-negative breast cancer patients (n = 430). Kaplan-Meier analysis showed that patients with a low percentage of Lewis^y/b ^positive cells (blue curve: <25% positive cells, n = 315) have significantly longer survival times than patients with >25% positive tumour cells (green curve: n = 115, p = 0.006).

Kaplan-Meier analysis of this cohort of breast cancer patients, with mean follow-up of 7 years, did not demonstrate significant differences in the overall survival between patients with a low percentage of Lewis^y/b ^positive cells (<25%) versus patients with >25% positive tumour cells in the cohort as a whole (Fig. 5). On subgroup analysis, however, a significant correlation (p = 0.006) was identified between the proportion of Lewis^y/b ^positive tumour cells and survival in lymph-node negative patients (Fig. 6). Analysis using the log-rank test showed that in these lymph-node negative tumours (n = 430), patients with a low percentage of Lewis^y/b ^positive cells (<25% positive cells, n = 315) have significantly longer survival times than patients with >25% positive tumour cells (n = 115, p = 0.006).

In contrast, there was no significant association between the intensity of Lewis^y/b ^expression and overall survival between the patient group with no expression of Lewis^y/b ^versus positive tumours (weak, moderate or strong staining) in the cohort as a whole (log rank = 0.667), or on subgroup analysis in lymph-node negative patients (log rank = 0.140).

Multivariate analysis, including the factors of tumour size, nodal status, tumour grade and Lewis^y/b ^expression showed that tumour size, tumour grade and nodal status were independent prognostic parameters, whereas Lewis^y/b ^expression was not an independent prognostic marker in this patient cohort.

## Discussion

Blood group related antigens are frequently altered in association with neoplastic transformation in many organs. This study illustrates the prognostic and potential predictive value of Lewis^y/b ^expression in a large series of 660 patients with invasive breast carcinoma. Forty-four percent of cases demonstrated Lewis^y/b ^expression, with eight percent showing extensive expression. A higher percentage of Lewis^y/b ^expression was significantly correlated with histological grade, NPI and histological tumour type group and, in lymph-node negative patients, overall survival decreased with increasing level of Lewis^y/b ^expression.

There are few previous reports of blood group antigens in relation to prognosis from breast cancer. Our results are in concordance with a previous study, however, in which higher expression of Lewis^a ^and Lewis^b ^was observed in lymph node negative tumours than in lymph node positive tumours, and higher expression of Lewis^b ^was seen in stage T4 than in stage T1 tumours, suggesting that the expression of sialosyl-Lewis^b ^and Lewis^a ^antigens in breast cancer may predict metastases to lymph nodes [16]. A positive correlation between Lewis^y ^expression and a poor prognosis has also been shown for superficial oesophageal carcinomas [17].

Lewis^y ^and Lewis^b ^are expressed on both glycolipids and glycoproteins [6,18]. It has been shown that an antibody, ABL-364, recognising Lewis^y ^can inhibit Erb-B signalling by rerouting these receptors to a submembrane compartment from which they rapidly recycle to the cell surface [19]. A previous study on this series of breast tissue arrays has shown that expression of the epidermal growth factor receptor (Erb-B1) was significantly associated with a high histological grade, high NPI score, negative ER status, larger tumour size, the development of distant metastases and death from cancer. We have also demonstrated that c-erbB-2 expression independently predicted for poor overall survival in this population of breast cancer patients [20]. There was no association, however, between Erb-B1 expression and Lewis^y ^expression in this group of tumours.

Lewis^y ^can be up-regulated in response to cellular stress. Indeed, up-regulation of Lewis^y ^in response to chemotherapeutic stress, and in particular to the chemotherapeutic agent 5-fluorouracil, has been previously reported [21]. In early stage breast cancer, expression of Lewis^y/b ^may, therefore, be a marker of aggressive or stressed tumours.

A range of Lewis^y ^antibodies have been identified, but these consistently cross react with Lewis^x ^and H type 2 structures. This can lead to undesirable cross reactivity with normal tissues and subsequent toxicity in clinical trials. In a phase I study of the murine BR55-2 anti-Lewis^y ^antibody, which cross reacts with H antigen in breast cancer patients, haematuria occurred in 6/12 patients and diarrhoea in 2/9 patients, with only transient reductions in skin lesions seen in 3 patients [22]. A chimeric BR-96-doxorubicin conjugate has also been evaluated in patients with a range of advanced cancers. BR-96 cross-reacts with B hapten and gastrointestinal binding was found to be dose limiting [23]. Finally, the Lewis^y ^specific humanised antibody 3S193 has shown good selectivity in binding studies and is about to enter phase I clinical trials [24]. Lewis^y ^has also been suggested as a target for cancer vaccines; however, immunising with synthetic Lewis^y ^generated carbohydrate specific antibodies but they failed to bind to tumour cells. In contrast, MMA383 is an anti-idiotypic antibody that mimics Lewis^y ^and stimulates good antibody responses. It has been humanised [25] and will shortly enter clinical trials.

## Conclusion

Analysis of Lewis^y/b ^expression in early stage breast cancer patients may aid selection of patients for more aggressive chemotherapy, as tumours with a high percentage of cells expressing Lewis^y/b ^appear to have a significantly greater chance of disease progression. This may also eventually allow stratification of patients for mAb therapy directed against these carbohydrate antigens; however, specific antibodies that directly induce cell killing but do not cross react with H or B blood groups first need to be selected. SC101/29 is a promising candidate and is currently being humanised for clinical trials.

## Abbreviations

ER = estrogen receptor; mAb = monoclonal antibody; NPI = Nottingham Prognostic Index.

## Competing interests

The authors declare that they have no competing interests.

## Authors' contributions

ZM and NFSW carried out the immunohistochemistry, scored the staining, analyzed the data and drafted the manuscript. TP made the SC101 primary antibody. LGD, IOE and IS designed the study and provided the tissue microarray. All authors were responsible for interpreting the results and drafting the article. All authors read and approved the final manuscript.

## Figures and Tables

**Table 1:** Patient and tumour characteristics

Tumour and patient characteristics	Proportion
Alive	86% (570)
Dead (of breast cancer)	14% (90)
Age (years)	
<40	9%(58)
41–50	29%(192)
51–60	33% (221)
>61	29% (189)
Menopausal status^a^	
Premenopausal	36% (227/623)
Postmenopausal	64% (396/623)
Histological grade	
Grade 1	20% (135)
Grade 2	33% (215)
Grade 3	47% (310)
Lymph node status	
Negative	65% (427)
Positive	35% (233)
Nottingham Prognostic Index^a^	
Good	35%(226/638)
Moderate	52%(332/638)
Poor	13%(80/632)
Tumour histological type^b^	
Group 1	5% (32/647)
Group 2	22% (142/647)
Group 3	12% (78/647)
Group 4	61% (395/647)
Vascular invasion^a^	
None or probable	69% (444/643)
Definite	31% (199/643)
Distant metastases	
Absent	85%(559)
Present	15% (101)
Any recurrence	
Absent	74% (488)
Present	26% (172)
Regional recurrence (axillary lymph node)	
Absent	91% (598)
Present	9% (62)
Local recurrence (in the breast)	
Absent	90% (591)
Present	10% (69)

^a^Percentage of total number of recorded cases. ^b^Tumour sections were classified into four prognostic type groups as previously described [26]: 1, excellent prognosis type (>80% 10 year survival) includes tubulo-lobular, tubular, mucinous and invasive cribriform carcinoma; 2, good types (60% to 80% 10 year survival) includes tubular mixed, mixed ductal with special type and alveolar lobular carcinoma; 3, moderate prognosis types (50% to 60% 10 year survival) includes classical lobular, medullary, atypical medullary and lobular mixed carcinoma; 4, poor prognosis types (≤ 50% 10 year survival) includes ductal/NST, solid lobular, mixed ductal and lobular carcinoma.

**Table 2:** Percentage of cells showing immunoreactivity of SC101

Percentage of SC101 positive cells	% of tumours
0% (no staining)	56 (n = 370)
1% to 25%	17 (n = 110)
25% to 50%	11 (n = 71)
5% to 75%	8 (n = 55)
>75%	8 (n = 54)
Total	660

**Table 3:** Intensity of SC101 expression

Immunohistochemical score	% of tumours
None	56 (n = 370)
Weak	16 (n = 106)
Moderate	13 (n = 89)
Strong	15 (n = 95)
Total	660

**Table 4:** Association of Lewis^y/b ^expression with clinicopathological characteristics (chi-square test)

Prognostic factors	Cut-off points	Intensity of staining (p-value)	Percentage of positive cells (p-value)
Age (years; median 56)	<40, 41–50, 51–60, >60	0.011	0.014
Menopausal status	Pre- or post-menopausal	0.547	0.917
Histological grade	Well, moderate, poor differentiated	0.009	<0.001
Lymph node (LN) stage	LN -/ LN+	0.368	0.419
Tumour size (mm)	<10, 11–20, 21–30, 31–40, 41–50	0.594	0.884
Nottingham Prognostic Index	Good, moderate, or poor	0.151	0.016
Tumour type	1, 2, 3 or 4	0.093	0.007
Vascular invasion	None or definite	0.293	0.316
Distant metastases	Absent or present	0.515	0.532
Any recurrence	Absent or present	0.533	0.743
Local recurrence	Absent or present	0.775	0.908
Regional recurrence	Absent or present	0.314	0.278

**Table 5:** Logistic regression analysis of percentage of Lewis^y/b ^positive cells^a^

Prognostic factors	Odds ratios (95% CI)	Test for trend (linear by linear)
Histological grade		<0.001
Well	1	
Moderate	1.51 (0.87–2.61)	
Poor	2.50 (1.51–4.16)	
Nottingham Prognostic Index		0.016
Good	1	
Moderate	1.63 (1.09–2.44)	
Poor	1.99 (1.13–3.50)	
Tumour type		0.007
Excellent	1	
Good	2.03 (0.66–6.23)	
Moderate	2.25 (0.701–7.25)	
Poor	3.12 (1.07–9.11)	

^a^Positive cells were categorized into two groups, high (<25%) and low (>25%). CI, confidence interval.
